# Changing our minds about changes of mind

**DOI:** 10.7554/eLife.14790

**Published:** 2016-03-07

**Authors:** Stephen M. Fleming

**Affiliations:** Wellcome Trust Centre for Neuroimaging, University College London, London, United Kingdomstephen.fleming@ucl.ac.uk

**Keywords:** decision making, motor control, computational neuroscience, metacognition, error detection, EEG, Human

## Abstract

Two theories that attempt to explain why we sometimes reverse a decision shortly after making it may both be correct.

**Related research articles** Murphy PR, Robertson IH, Harty S, O’Connell RG. 2016. Neural evidence accumulation persists after choice to inform metacognitive judgments. *eLife* **4**:e11946. doi: 10.7554/eLife.11946; van den Berg R, Anandalingam K, Zylberberg A, Kiani R, Shadlen MN, Wolpert DM. 2016. A common mechanism underlies changes of mind about decisions and confidence. *eLife* **5**:e12192. doi: 10.7554/eLife.12192

**Image** Electrical activity in the brain during a decision-making task

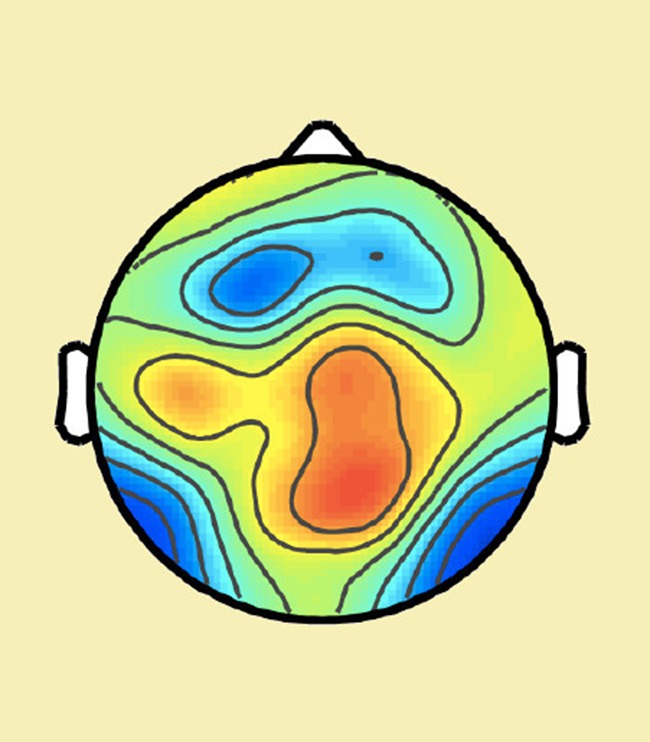

Benjamin Franklin once quipped that “There are three things extremely hard: steel, a diamond, and to know oneself”. A simple form of self-knowledge is recognizing that a previous choice was incorrect without receiving any feedback on that decision (Rabbitt, 1966). Every decision we make, from detecting a faint sound to choosing a new job, comes with a degree of confidence that we have made the right call. If this confidence level is sufficiently low we might change our minds and reverse our choice. Theoretical models and experimental work both suggest that decision making operates by accumulating evidence for or against a particular option (Gold and Shadlen, 2007). But whether similar evidence accumulation mechanisms also underpin changes of mind has remained unclear.

There are two schools of thought on this problem. One suggestion is that changes of mind happen because we continue to weigh evidence after a choice has been made (Resulaj et al., 2009); this process is called post-decision evidence accumulation. An alternative idea is that the brain uses additional mechanisms to detect and correct previous errors. Support for this theory comes from findings that show that error-related signals are produced in the medial frontal cortex of the human brain (Cavanagh and Frank, 2014; Gehring et al., 1993). People who have damage to the frontal regions of the brain are also unable to “self-monitor” and identify errors they have made without external feedback (Ham et al., 2013). Now a pair of studies in eLife provides the most detailed account yet of the mechanisms underpinning changes of mind – and together, they indicate that both ideas could be right.

Both studies asked human volunteers to perform a rapid series of judgments about what they saw on a computer screen. In one of the studies Daniel Wolpert, Michael Shadlen and colleagues – including Roland van den Berg as first author – asked volunteers to decide whether a patch of flickering dots was drifting to the left or right (van den Berg et al., 2016). The volunteers indicated their choice by moving a handle in the corresponding direction and simultaneously indicated their confidence in their decision by moving the handle to an upper or lower target. This allowed the researchers – who are based at the University of Cambridge, Columbia University and New York University – to track how each decision evolved over time and to observe occasional swerves from one target to the other that they labelled as changes of mind or changes of confidence.

Each volunteer performed several thousand judgments at different motion strengths (which are determined by the proportion of coherently moving dots). A previous paper found that the strength of the accumulated evidence, together with the elapsed time, could predict how confident the volunteers felt when they made their decision (Kiani et al., 2014). By extending this model to allow evidence to continue to accumulate after the decision had been made, van den Berg et al. could also explain changes of mind and changes of confidence through a common mechanism. Both the extended model and the volunteers were more likely to switch from low to high confidence at the last second, particularly when the motion was strong.

What is the neural basis of post-decision evidence accumulation? This question was tackled by Peter Murphy of Trinity College Dublin and Leiden University and colleagues in the other paper (Murphy et al., 2015). While wearing electroencephalography (EEG) caps, human volunteers were asked to press a button each time a colour word such as “red” appeared on a computer screen. However, they were instructed to not press the button if either the same word was repeated twice or if the meaning of the word and the font colour matched (for example, “red” written in red text). This is a difficult task to perform quickly, and mistakes were made on 43% of the trials on average. To assess self-monitoring, the volunteers pressed a separate button if they noticed making an error.

The analysis of the brain activity detected in the EEG recordings focused on an electrical signal called the centroparietal positivity. This is a human equivalent of the evidence accumulation signals previously recorded from the brains of monkeys as they made simple decisions. (O'Connell et al., 2012). Murphy et al. found that this signal ramped up to a threshold level during both pre- and post-decision evidence accumulation, and predicted the probability and timing of error detection. This finding indicates that the same evidence accumulation system is engaged both when making a decision about external events and when deciding whether one’s own previous decision is wrong.

Intriguingly, Murphy et al. also found an additional electrical signal in the frontal cortex – an oscillation in a range of low frequencies called the theta band – that was selectively engaged when errors were detected. This signal influenced how much impact the post-decision evidence accumulation signal had on changes of mind. This finding suggests – in line with early work on error monitoring – that a “quick and dirty” error signal in the frontal cortex may subsequently trigger post-decision evidence accumulation to work out whether a change of mind is warranted.

While both papers confirm the importance of post-decision evidence accumulation for self-monitoring, they diverge on the source of this evidence. van den Berg et al. suggest that a single “bottom up” stream of evidence is continually accumulated both before and after a choice: some of this evidence is available at the time the decision is made but, due to processing delays, does not influence the initial decision. In contrast, Murphy et al. suggest that top-down signals – information that feeds back to influence earlier stages of processing – provide an additional input to the post-decision evidence accumulation process (Figure 1).

**Figure 1.**
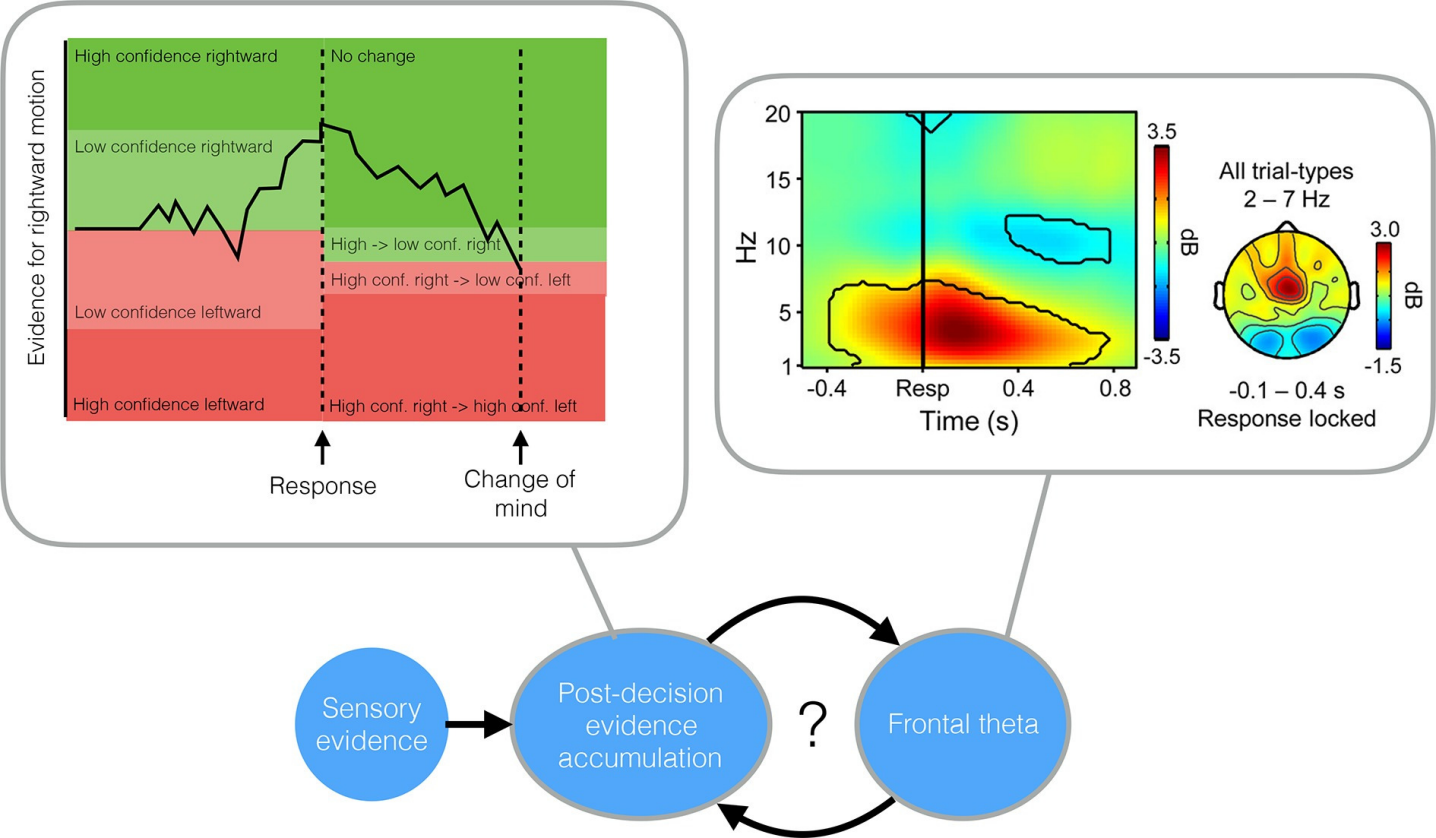
Two studies suggest that evidence accumulated after a decision can cause a change in mind. Top left: The evidence accumulation model used by van den Berg et al. can explain both choices and changes of mind. The black trace shows the accumulated evidence for a rightward or leftward motion response. Stronger evidence is associated with higher confidence. After the response is made (in this case, a high confidence rightward response) evidence continues to accumulate, leading to a reversal of the choice (a low confidence, leftward response). Top right: A time-frequency plot of EEG activity recorded by Murphy et al. (2015) (their Figure 3a). Recordings from frontocentral brain regions revealed an increased theta oscillation on trials in which subjects detected their own errors. This oscillation influenced post-decision evidence accumulation. Bottom: A possible mechanism that incorporates the findings of both van den Berg et al. and Murphy et al.: post-decision evidence accumulation may integrate both bottom-up signals from external sensory evidence and top-down signals in the form of the theta oscillations.

Differences in the design of the two studies may limit the extent to which we can compare and combine their findings. In particular, Murphy et al. asked volunteers to report errors with a second button press, whereas changes of mind were registered continuously in van den Berg et al.’s study. The contribution of the theta signal from the frontal cortex may be more important in the former case (when an overt error has already been committed) than the latter. Future research could combine the approaches taken in these two papers. For instance, it would be of interest to monitor EEG during experiments like those performed by Wolpert et al. to establish the relative contributions of bottom-up and top-down influences on changes of mind.

Psychologists have long been interested in metacognition – the ability to reflect on and evaluate our own thoughts and behaviour. The neural basis of metacognition is likely to be complex and multi-faceted, but these papers suggest that mechanisms supporting simpler types of decision-making may have been co-opted for self-monitoring. Over 200 years after Benjamin Franklin’s death, we now know that steel and diamonds are constructed from simpler atomic and molecular building blocks. By studying the dynamics of simple decisions we may eventually uncover the components of his third hard substance, self-knowledge.
